# Supplementary material for: Formation of neural networks with structural and functional features consistent with small-world network topology on surface-grafted polymer particles
Source: R Soc Open Sci. 2019 Oct 23;6(10):191086. doi: 10.1098/rsos.191086 (PMC6837210; doi:10.1098/rsos.191086)

**Supplementary information**

**Supplementary Figure 1. Immunocytochemistry demonstrating the presence of neural lineage markers in the neural networks developed on the surface-grafted polymer particles (PPs).** Following 1 month of differentiation and maintenance of the rat NSCs on the PPs, immunocytochemistry confirmed the presence of differentiated cells attached to the surface of the particles. **A,B**) Fluorescently labelled single cells attached to the surface of the PPs. The anti-CNPase antibody (red) shows oligodendrocytes, while anti-GFAP antibody (green) shows astrocytes and other glial cells. Anti-MAP2 staining is also consistent with the presence of cells of a neuronal fate in **B** (magenta). The single-color channels are merged and overlaid by a brightfield image of the same area. **C**) Fluorescently labelled cells from the same sample attached to the well-plate surface. 100μm scalebars.


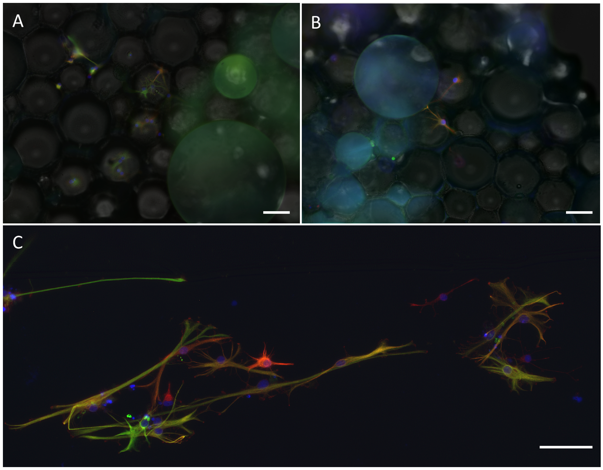

Supplement: Immunocytochemistry demonstrating the presence of neural lineage markers in the neural networks developed on the surface-grafted polymer particles (PPs). [file rsos191086supp1.docx]
